# The role of maintenance therapy in multiple myeloma

**DOI:** 10.1038/bcj.2016.89

**Published:** 2016-10-21

**Authors:** B Lipe, R Vukas, J Mikhael

**Affiliations:** 1 Department of Hematology, University of Rochester, Rochester, NY, USA; 2 Department of Biostatistics, University of Kansas Medical Center, Kansas City, KS, USA; 3 Department of Medicine, Mayo Clinic Arizona, Phoenix, AZ, USA

## Abstract

Multiple myeloma is the second most common type of blood cancer and remains incurable despite advances in therapy. Current therapy for multiple myeloma includes a phased-approach, often consisting of initial induction therapy, consolidation and maintenance therapy. With an ever-growing landscape of treatment options, the approach to optimal therapy has become increasingly complex. Specifically, controversy surrounds the optimal use and duration of maintenance therapy. We conducted a comprehensive literature search to analyze the most current literature and to provide recommendations for maintenance therapy in multiple myeloma.

## Introduction

Multiple myeloma (MM) is an incurable hematologic malignancy of plasma cells with a median overall survival (OS) of 6.1 years for patients diagnosed since 2006.^1^ The disease is characterized by periods of active disease requiring systemic therapy followed by periods of relative quiescence dependent on both biology and ongoing treatment. Phases of treatment for MM have been characterized as induction therapy (a limited period of therapy for initial disease reduction), consolidation therapy (more limited therapy aimed at deepened therapeutic response) and maintenance therapy (continuous therapy aimed at long term disease control). This review will focus on maintenance therapy and assume patients have received optimal and appropriate initial and consolidation therapy, which currently consists of two or three drug combinations depending on the clinical context^2,3,4, 5^

Maintenance therapy aims to extend the period of disease quiescence through continued treatment and to thereby extend progression-free survival (PFS) and OS.^6^ Because the treatment is administered for a prolonged period of time, particular emphasis is placed on the tolerability and toxicity of maintenance therapy. The concept of maintenance therapy in MM has been around for over 30 years, but treatment toxicity has limited the applicability of maintenance therapy until the relatively recent introduction of newer therapies. Interferon therapy was heavily studied and two meta-analysis demonstrated an improvement in OS of 4 and 7 months, respectively.^7, 8^ However, there is little data to guide which specific patients may receive benefit from interferon maintenance and toxicity is high with decreased quality of life (QOL),^9^ limiting the practical utility of interferon maintenance. Corticosteroids have been investigated as a maintenance therapy, but there is conflicting data regarding the efficacy of corticosteroids as maintenance^10, 11^ and concern for increased drug resistance at relapse.^12^ Thalidomide has been heavily studied as a maintenance drug in MM, with improvements in PFS but conflicting results on OS. One meta-analysis showed a late OS benefit, but raised concerns about the use of thalidomide as maintenance therapy in patients with high risk disease by cytogenetics.^13^ Thalidomide maintenance has also demonstrated significant toxicity, primarily neurotoxicity. In the medical research council IX study, 52% of patients discontinued thalidomide maintenance after a median duration of 7 months.^13^ Another large study failed to show improvement in OS with thalidomide maintenance, but did show a decrease in QOL.^14^ Given the challenges with toxicity, thalidomide has largely been abandoned as maintenance therapy in MM in newly designed trials and will therefore not be the focus of this review.

The advent of first generation novel agents, including lenalidomide and bortezomib offer reduced toxicity and the opportunity to revisit the concept of maintenance therapy. More recently, the landscape of MM therapy continues to expand further with the approval of next generation proteasome inhibitors and immunomodulatory agents as well as the recent approval of new drugs with novel mechanisms of action including monoclonal antibodies and histone deacetylase inhibitors.^15, 16, 17^ With the rapid changes in MM therapy, our goal with this review is to analyze the current literature and provide recommendations for maintenance therapy in MM, primarily with lenalidomide and bortezomib.

## Methods

A comprehensive literature search was conducted by a professional librarian to identify relevant published literature and clinical studies regarding the management of chemotherapy for multiple myeloma. The following databases were searched: PubMed, The Cochrane Library, ClinicalTrials.gov, PubMed Health and Web of Science. Embase was not available to the authors. The entire years of coverage of each database was searched through March 2016. The main search strategy combined the keywords and Medical Subject Headings terms of multiple myeloma or plasma cell neoplasms and maintenance chemotherapy or chemotherapy management. Case reports and non-English language items were excluded from the results. This search strategy resulted in 1055 unique items. The results of each database were:

PubMed: 375

PubMed Health: 3

Cochrane Library: 16

ClinicalTrials.gov: 212

Web of Science: 449

## Study selection

Title and abstracts were reviewed and included for analysis if they were in English, peer-reviewed, and discussed the use of lenalidomide or bortezomib for maintenance therapy. Articles discussing only thalidomide, corticosteroids, or interferon maintenance, or review articles were excluded. Articles meeting the above criteria were reviewed in full with the following data extracted: phase of trial, number of patients, treatment regimen including prior transplantation, age of patients, duration of therapy and efficacy data including response rates, OS and PFS when available. The quality of randomized studies was assessed using the Jadad score,^18^ (Table 1). Quality of evidence and recommendation were graded based on Grading Recommendations Assessment Development and Evaluation tool.^19^

## Results

**Figure 1:**
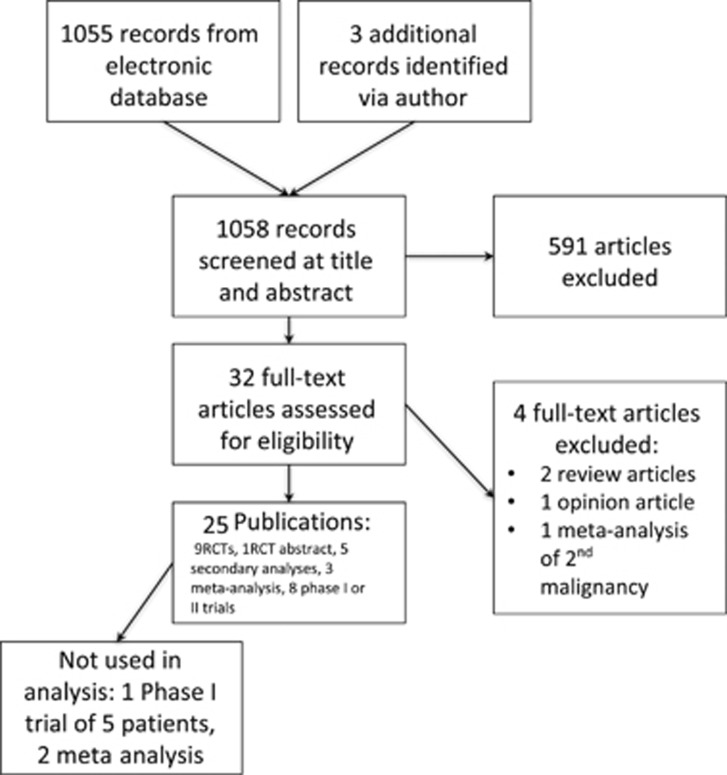
Selection of included articles.

A total of 26 studies were included: 9 randomized controlled trials (RCTs), 1 RCT in abstract form, 5 secondary analyses presenting data from RCTs, 3 meta-analysis and 8 treatment trials Phase I or II (Figure 1). Five RCT and four treatment trials involved the use of bortezomib, whereas two treatment trials involved the use of both lenalidomide and bortezomib. Four RCTs included the use of autologous stem cell transplant (ASCT), six treatment trials included the use of ASCT and one included the use of allogeneic transplant (Figure 1).

### Transplant ineligible

For elderly or transplant ineligible patients with newly diagnosed MM, we evaluated the use of lenalidomide maintenance therapy. MM-015^(ref. 20)^ was a randomized, double-blind, placebo-controlled trial of patients over 65 years of age comparing melphalan, prednisone and lenalidomide (MPR) to MPR with lenalidomide maintenance (MPR-R) to melphalan and prednisone (MP). Patients with progressive disease were allowed to enroll in an extension phase of the trial to receive lenalidomide or lenalidomide plus dexamethasone. The study showed an improvement in PFS for patients <75 treated on the MPR-R arm along with greater and more persistent improvements in health-related QOL scores, though there was no difference in OS amongst the arms.^21^ The FIRST trial was also a three-arm trial comparing continuous lenalidomide and dexamethasone to fixed dose lenalidomide dexamethasone or melphalan, prednisone and thalidomide (MPT).^22^ The study demonstrated improved PFS and OS for patients treated with continuous lenalidomide and dexamethasone, including those >age 75. Subsequent analysis demonstrated improved health-related QOL for patients treated with lenalidomide and dexamethasone vs MPT.^23^ The recent studies E1A06 and HOVON 87/NMSG 18 have compared thalidomide maintenance to lenalidomide-based maintenance.^24, 25^ Both trials randomized patients to melphalan, prednisone and thalidomide with thalidomide maintenance (MPT-T) vs MPR-R. Both trials demonstrated similar PFS and OS between the groups, but QOL was improved on the MPR-R group in E1A06 and neurotoxicity and drop-out rates were lower with MPR-R for the HOVON trial. The combined data from these trials provide evidence of the improved tolerability for lenalidomide maintenance therapy vs thalidomide.

In the transplant ineligible setting, randomized trials have looked at the role of maintenance therapy incorporating bortezomib. In the GEM2005MAS65 trial, patients were randomized in a 2x2 design to either bortezomib/melphalan/prednisone (VMP) or bortezomib/thalidomide/prednisone (VTP) for induction and consolidation.^26^ Patients were then randomized to maintenance therapy with bortezomib/prednisone (VP) vs bortezomib/thalidomide (VT). The study showed that maintenance therapy improved the depth of response from induction therapy and demonstrated a median PFS of 32 months. For patients improving on their initial responses, PFS and OS were increased. Although there was a trend to increased PFS and OS for the VT maintenance vs VP arm, this did not reach statistical significance. In GIMEMA MM03-05, patients were randomized to VMP plus thalidomide (VMPT) followed by 2 years of maintenance VT vs VMP without maintenance.^27^ The study demonstrated an improvement in PFS and OS for the VMPT–VT arm with the median OS from time of relapse similar in each arm. The UPFRONT trial was a community-based study comparing VD vs VMP vs VTD plus V maintenance.^28^ The study showed that VD was more tolerable than VMP or VTD during induction, and there was no difference in PFS amongst the arms. There was minimal increase in toxicity during maintenance suggesting that the improved tolerability of maintenance therapy may overcome any differences amongst the treatment regimens.

### Transplant eligible

Results are available from randomized trials of lenalidomide maintenance in patients who have received ASCT. The IFM 2005-02 trial randomized patients after induction, ASCT and lenalidomide consolidation for two cycles to lenalidomide maintenance vs placebo.^29, 30^ Patients on the placebo arm were not allowed to crossover at unblinding. Lenalidomide maintenance was stopped early for a risk of second cancers, and showed an improvement in PFS, but not OS for patients treated with lenalidomide maintenance. Cancer and Leukemia Group B (CALGB) 100104 randomized patients 100 days after ASCT to lenalidomide maintenance vs placebo.^31, 32^ The study demonstrated improved PFS and OS for the lenalidomide arm despite allowing crossover from the placebo arm to the lenalidomide arm at unblinding. This study did not discontinue lenalidomide maintenance despite an increased risk for second cancers. Many patients on the trial had received lenalidomide as induction therapy and this was associated with improved time to progression on the lenalidomide maintenance arm. The results were maintained for patients in complete response (CR) and those with less than a CR after transplant.^32^ RV-MM-P1209 was a 2 × 2 randomized factorial design.^33^ Patients were given lenalidomide and dexamethasone induction then randomized to tandem-ASCT vs MPR consolidation with or without lenalidomide maintenance therapy. The study demonstrated improved PFS for the lenalidomide maintenance groups, regardless of consolidation regimen. There was no difference in OS or the rate of second primary malignancies (SPM). A recent meta-analysis of these three trials of maintenance lenalidomide therapy after ASCT demonstrated improved OS for patients receiving lenalidomide maintenance, median OS not reached vs 86 months. The improvement in OS was preserved for patients with⩽PR and those with CR/very good partial response (VGPR).^34^

A sequential use of novel agents was investigated in a newly diagnosed population in a phase II trial.^35^ In this trial, patients were treated with bortezomib, PEGylated liposomal doxorubicin, dexamethasone induction followed by ASCT, lenalidomide and prednisone consolidation and lenalidomide maintenance therapy until progression. The study demonstrated an improvement in depth of response with maintenance therapy I.

Bortezomib maintenance for transplant-eligible patients has been evaluated in the phase III HOVON-65/GMMG trial.^36, 37^ The study randomized patients to vincristine/doxorubicin/dexamethasone (VAD) with thalidomide maintenance vs bortezomib/doxorubicin/dexamethasone (PAD) with V maintenance × 2 years. The study demonstrated improved PFS and OS for the PAD arm with an improved benefit seen in high risk patients with elevated creatinine or deletion 17p13.

Combined lenalidomide and bortezomib maintenance was studied in patients with high-risk multiple myeloma.^38^ In this study, patients received ASCT followed by up to 3 years of maintenance therapy with bortezomib, lenalidomide and dexamethasone maintenance followed by indefinite lenalidomide maintenance. A total of 45 patients were treated in this trial and demonstrated a median PFS of 32 months with a 3-year OS of 93%. A phase 1 study, S1211, has also reported on the safety of maintenance therapy in patients with newly diagnosed high risk myeloma.^39^ The trial examined the use of lenalidomide, bortezomib, elotuzumab and dexamethasone as maintenance therapy, and suggests little additive toxicity with the incorporation of elotuzumab to lenalidomide and bortezomib based therapy. The phase II results of this trial are upcoming.

## Discussion

Among the trials, bortezomib and lenalidomide were used with different dosing, frequency and combination strategies amongst different studies making comparisons difficult and limiting the strength of any single recommendation. Despite the variability, there is strong evidence to suggest that maintenance therapy improves PFS. Data regarding OS is more variable, but at least three trials suggest improved OS with maintenance therapy.^22, 27, 31^ Other major considerations of maintenance therapy revolve around concerns for toxicity including second malignancies, optimal duration of therapy and selection of agent specifically with regard to depth of response and disease risk stratification, and cost of therapy.

## QOL/toxicity

Several studies have specifically looked at QOL in transplant ineligible patients undergoing maintenance therapy and provide evidence that QOL can be improved or at least maintained in the transplant ineligible population.^21, 22, 28^ In the lenalidomide trials, therapy, including maintenance, was generally well tolerated with the main side effects of hematologic toxicity, infection and rare thrombotic events.^20, 22^ During maintenance therapy, there was not a significant rate of progressive hematologic toxicity, though infection rates were still slightly increased in the FIRST trial. Amongst the bortezomib trials, toxicities were as expected with hematologic toxicity and neuropathy the most common complications. VTP combinations were associated with higher rates of complications including infections and cardiac events, resulting in dosage adjustments and therapy discontinuation amongst all the studies. In the MM03-05 study, this was primarily restricted to toxicity during induction therapy. Although the UPFRONT study was expected to show inferiority for the doublet combination of VD vs the triplet combinations, this was likely not seen because of the improved tolerability of the doublet regimen with tolerability maintained during maintenance therapy.

In the transplant eligible setting, toxicity is also of concern. In the lenalidomide trials, the treatment was overall tolerable with relatively low rates of discontinuation of therapy for toxicity. Hematologic toxicity was more common in the lenalidomide maintenance arms across the trials. IFM 2005-02 showed higher rates of thromboembolic complications for the lenalidomide maintenance patients, whereas the RV-MMP1209 trial showed higher rates of infection and dermatologic complications. In the HOVON-65 trial, 11% of patients stopped bortezomib maintenance therapy early with neuropathy developing during maintenance in 5% of patients. It should be noted that bortezomib was administered via IV during the study. Subsequent data have suggested decreased toxicity with subcutaneous administration, but the impact of SC administration on toxicity in maintenance therapy is unknown.

## Duration of therapy

The optimal duration of maintenance therapy is currently controversial. There is concern about the rate of second malignancy for lenalidomide containing maintenance regimens. The CALGB, IFM and MM-015 trials reported an increased risk of second malignancies with the use of lenalidomide maintenance therapy (10.8 vs 4.4%, 2.3 vs 1.3%, 13 vs 4% respectively). Although the IFM trial was initially planned for treatment until disease progression, maintenance was capped at 2 years because of the increased rate of second cancers. Development of a second cancer with lenalidomide appears to be the result of a synergistic effect with alkylator therapy as the FIRST trial did not show an increased risk of second cancers with continuous lenalidomide in the absence of alkylator therapy. The median time to development of a second cancer was 15 months after the start of maintenance therapy as reported in the CALGB study, and there are no data to suggest that capping maintenance therapy at 2 years actually reduces the risk of second malignancies. On the contrary, PFS is improved across trials with lenalidomide therapy, even when accounting for the incidence of second cancers. Although individual trials have reported an increased risk of second primary malignancy (SPM), a population based analysis examined the risk of SPM for patients before and after the advent of novel agents and found no increase in rates of SPM after the advent of novel agents.^40^

Duration of maintenance therapy has also been limited to 2 years in some studies because of a concern that ongoing therapy selects for a more resistant disease at the time of relapse. Speculation exists regarding the OS advantage seen in the CALGB trial that was not seen in the IFM 2005-02 trial, despite the ability of control patients in the CALGB trial to cross-over to lenalidomide at the time of disease relapse. One important difference amongst the trials was the use of consolidation lenalidomide amongst both arms in the IFM trial and the early use of lenalidomide in induction for the CALGB trial. Another finding from the IFM trial showed a decrease in the duration of PFS2 for patients who relapse on maintenance therapy compared with patients who relapse but were not on maintenance therapy.^41^ This decrease in PFS2 has been attributed to a more resistant MM clone that is harder to treat because it emerges in the setting of ongoing therapy. However, analysis of other maintenance trials has not shown a decrease in PFS2^(refs 42, 43^) and an OS survival advantage has been maintained in the CALGB and FIRST trials with ongoing maintenance therapy that was not capped. A recent pooled analysis of three trials demonstrated improved PFS1, PFS2 and OS for patients receiving continuous therapy vs fixed dose therapy.^44^ Given the encouraging data that prolonged PFS1 does not limit PFS2 and that treatment options after PFS2 are expanding given the newly available and upcoming therapies, we do not advocate capping maintenance therapy at 2 years for most patients.

## Choice of agent

Although there is encouraging data that bortezomib improves PFS, there is now evidence that lenalidomide maintenance improves OS. Although some argue for a sequential approach to therapy, there is no data to demonstrate an advantage to this approach. Alternatively, both the HOVON and CALGB trials suggest improved PFS with the early incorporation of novel agents that are then used as maintenance. In addition, the early incorporation of agents into induction therapy will ensure therapeutic efficacy as maintenance therapy is considered. Although lenalidomide should be considered the standard of care for standard risk patients, bortezomib might be considered for patients with high risk disease or for those intolerant or resistant to lenalidomide. Improvement in OS has been seen with the use of bortezomib for patients with high risk disease including 17p deletion in the HOVON trial, but OS data for patients in the lenalidomide trials is pending. Although there is limited data on combination therapy for maintenance in the newly diagnosed setting, combination therapy after limited single ASCT has demonstrated encouraging OS data.^38^

## Cost

Cost of therapy is of consideration when planning maintenance therapy. A cost analysis comparison of lenalidomide vs bortezomib was done in Canada and showed decreased cost with bortezomib ($32 560 per year vs $144 976 per year for lenalidomide).^45^ However, the Canadian cost analysis examined cost from a health payer perspective and did not consider the economic or QOL impact on patients receiving oral therapy that can be administered at home (lenalidomide) vs weekly injection therapy that must be administered at a care facility (bortezomib). In the United States, patients on Medicare have additional cost considerations depending on their supplemental insurance. Payment assistance plans can often offset the out of pocket expenses to patients for oral therapy, but this is unique to each patient. Ultimately, the economic and personal cost considerations of lenalidomide vs bortezomib based therapy need to be considered on an individual patient basis. With new therapies, the mode of medication administration may become less relevant.

## Future options

Looking forward, the approval of new drugs continues to change the landscape of myeloma therapy. Options for salvage therapies are increased for relapsed patients. Currently, trials are incorporating the use of pomalidomide, carfilzomib and the monoclonal antibodies into ongoing maintenance therapy (clinicaltrials.gov) while attempting to identify patient and disease-specific predictors of response.^46^ Results of these trials will expand our experience and knowledge base while provoking new questions, concerns and recommendations. Ultimately, we continue to make progress in the treatment of MM and look forward to increasingly complex data regarding optimal care.

## Recommendations

**Figure 2:**
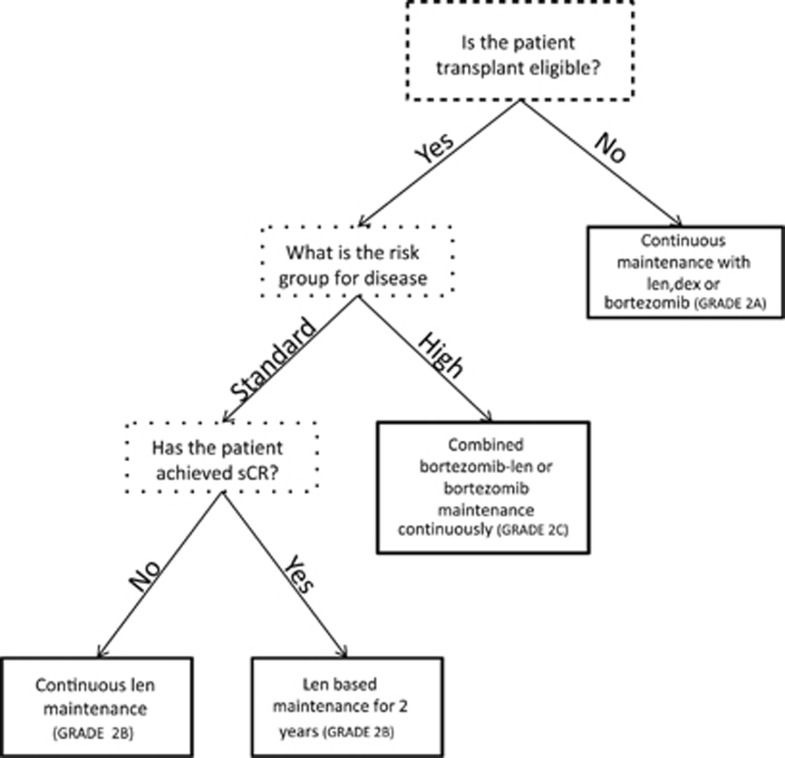
Flowchart for maintenance recommendations.

In a transplant ineligible population, we argue that increased PFS is a worthwhile goal if QOL is maintained and can delay the onset of disease side effects (Figure 2). As the majority of trials show a PFS advantage and the FIRST trial showed an OS survival advantage for continuous maintenance therapy, we recommend continuous maintenance therapy with lenalidomide/dexamethasone or bortezomib for all patients (GRADE 2A). We recommend the choice of maintenance therapy be matched to the induction regimen. For patients receiving both lenalidomide and bortezomib during induction, we recommend the choice of maintenance therapy be guided by patient preference, toxicity profile and risk-stratification of disease.

For transplant eligible patients, we would recommend stratified maintenance therapy based on risk features and depth of response. For standard risk patients who have achieved a sustained sCR, we would consider lenalidomide maintenance therapy for 2 years (GRADE 2B). Although this may be a controversial recommendation, there may be a subset of patients in CR who will not have disease relapse.^47^ Technology is improving and our understanding of CR is evolving, but we are currently unable to predict which patients with CR will never relapse. Given the side effects and potential long-term toxicity, impact on QOL and patient cost, we believe patients in maintained stringent CR after 2 years can consider stopping maintenance therapy.

For patients with less than a CR, we would recommend indefinite maintenance therapy with lenalidomide (GRADE 2B). We would not recommend maintenance with a drug for which a patient is known to be refractory. If patients are intolerant or resistant to lenalidomide, bortezomib maintenance should be used (GRADE 2B). As bortezomib maintenance has shown an advantage for patients with high risk disease via cytogenetics and combined maintenance has demonstrated promising PFS and OS results, we would advocate for a combined bortezomib–lenalidomide or bortezomib based maintenance strategy for high risk patients (GRADE 2C).

## Figures and Tables

**Table 1:** List of randomized controlled trials used in analysis

*Trial (ref. no.)*	*Schema*	*Maintenance dosing*	*Age (# pts enrolled)*	*Median PFS vs comparator*	*RR vs comparator*	*OS vs comparator*	*Median follow up*	*PFS2*	*Rate of second malignancy*	*QOL change*	*JADAD*
MM-015^(refs 20, 21)^	MPR-R vs MPR vs MP induction for 9 cycles	Len 10 mg for 21/28 days to progression	65 or older, transplant ineligible (459)	31 vs 14 vs 13 mos	77% vs 68% vs 50%	Median OS 54 vs 52 vs 55 mos	53 mos	39.7 mos (MPR-R) vs 28.5 mos (MP)	13% vs 10% vs 4% at 4 yrs	↑MPR-R	3
FIRST^22, 23^	RD continuous vs RD 17 cycles vs MPT 72 weeks	Len 25 mg 21/28 days and dex 40 mg weekly to progression	65 or older or transplant ineligible (1623)	25.5 vs 20.7 vs 21.2 mos	75% vs 73% vs 62%	Median OS 58.9 vs 56.7 vs 48.5 mos	45.5 mos	42.9 vs 40 vs 35 mos	3% vs 6% vs 5%	↑RD	2
E1A06^(ref. 24)^	MPT-T vs mPR-R induction for 12 cycles	Thal 100 mg daily vs Len 10 mg daily to progression	65 or older or transplant ineligible (306)	21 vs 18.7 mos	Adjusted 75.3 vs 70.4	Median OS 50.6 vs 47.74 mos	40.7 mos		3.47 vs 2.01 cases/100 person years	↑mPR-R	3
HOVON 87/NMSG 18^(ref. 25)^	MPT-T vs MPR-R induction for 9 cycles	Thal 100 mg daily vs Len 10 mg days 1–21 of 28 days	65 or older or transplant ineligible (637)	20 vs 23 mos	81% vs 84%	52% vs 56% at 4 yrs	36 mos		7% vs 6%		3
GEM-2005MAS65^(ref.26)^	VMP vs VTP × VT vs VP	Bor 1.3 mg/m^2^ days 1,4,8,11 q 3 mos+T 50 mg daily or P 50 mg qod for up to 3 yrs	65 or older (260)	32 vs 23 mos	After maintenance Cr 44% vs 39%	No difference in maintenance arms	62 mos		2% vs 2%		5
GIMEMA MM03-05^(ref. 27)^	VMPT–VT vs VMP	Bor 1.3 mg/m2q 15 days+T 50 mg dailyx2 yrs	Transplant ineligible (511)	35.5 vs 24.8 mos	Rate or CR 38% vs 24%	5 yrs OS 61% vs 51%	54 mos	OS after relapse, no difference			3
UPFRONT^28^	VD-V vs VTD-V vs VMP-V	Bor 1.6 mg/m^2^ 4/5 weeksx25 weeks	Transplant ineligible (502)	14.7 vs 15.4 vs 17.3 mos	73% vs 80% vs 70%	Median 49.8 vs 51.5 vs 53.1	42.7 mos	Median time to third line therapy 30.9 vs 38.5 vs 36.3 mos	1 patient on VMP arm	↓ QOL with induction all arms	
IFM 2005-02^(refs 29, 30)^	Post ASCT len consolidation then Len maintenance vs placebo	Len 10 mg dailyx3 mos then 15 mg daily capped at 2 yrs	<65 yrs and ASCT within 6 mos (614)	41 vs 23 mos	Rate of CR or VGPR 27% vs 25%	5 yrs OS 68% vs 67%	70 mos	10 vs 18 mos	2.3 per 100 patient years vs 1.3		
CALGB 100104^(refs 31, 32)^	D100 post ASCT Len maintenance vs placebo	Len 10 mg dailyx3 mos, then 15 mg daily to progression	<71 yrs and 100 days post ASCT (460)	Median TTP 53 vs 27 mos		Not reached for maintenance arm vs 76 mos	65 mos	No difference in OS after disease progression	10.8% vs 4.4%		
RV-MM-P1209^(ref. 33)^	(2 × 2 design) RD followed by ASCT × 2 vs MPR followed by len vs no len maintenance	Len 10 mg 21/28 days to progression	<65 yrs (273)	41.9 vs 21.6 mos	Rate of CR with maintenance 35.5% and 33.8% vs 15.7% (post ASCT) and 20% (post MPR)	3 yrs OS 88% vs 79.2%	51.2 mos	SPM in 11 patients, 5 during len maintenance			
HOVON-65/GMMG-HD4^(refs 36, 37)^	VAD+ASCT+Thal maintenance vs PAD+ASCT+Bor maintenance	T hal 50 mg daily vs Bor 1.3 mg/m^2^ qow for 2 yrs	<65 yrs (827)	28 mos vs 35 mos	Rate of CR+nCR 34% vs 49%	5 yrs OS 555 vs 61%	67 mos	5% vs 3% at 5 yrs			

Abbreviations: ASCT, autologous stem cell transplant; CALGB, Cancer and Leukemia Group B; CR, complete response; mos, months; MPR, melphalan, prednisone and lenalidomide; MPR-R, melphalan, prednisone and lenalidomide with lenalidomide maintenance; MPT, melphalan, prednisone and thalidomide; MPT-T, melphalan, prednisone and thalidomide with thalidomide maintenance; nCR, near complete response; OS, overall survival; PAD, bortezomib/doxorubicin/dexamethasone; PFS, progression-free survival; QOL, quality of life; RD, lenalidomide dexamethasone; RR, response rate; SPM, second primary malignancy; TTP, time to progression; VAD, vincristine/doxorubicin/dexamethasone; VD-V, bortezomib dexamethasone (induction)- bortezomib (maintenance); VGPR, very good partial response; VMP, bortezomib/melphalan/prednisone; VMP-V, bortezomib melphalan dexamethasone (induction)- bortezomib (maintenance); VMPT, VMP plus thalidomide; VP, bortezomib/prednisone; VT, bortezomib/thalidomide; VTD-V, bortezomib thalidomide dexamethasone (induction)- bortezomib (maintenance); VTP, bortezomib/thalidomide/prednisone; yrs, years.

## Notes

Brea Lipe: Consultancy for Takeda; Joseph Mikhael: Institutional research funding from Abbvie, Celgene, Onyx and Sanofi; and Rachel Vukas declare no conflict of interest.
